# Factors Associated With Poor Glycemic Control or Wide Glycemic Variability Among Diabetes Patients in Hawaii, 2006–2009

**DOI:** 10.5888/pcd9.120065

**Published:** 2012-09-27

**Authors:** Deborah Taira Juarez, Tetine Sentell, Sheri Tokumaru, Roy Goo, James W. Davis, Marjorie M. Mau

**Affiliations:** Author Affiliations: Tetine Sentell, James W. Davis, Marjorie M. Mau, John A. Burns School of Medicine, Honolulu, Hawaii; Sheri Tokumaru, Roy Goo, University of Hawaii at Hilo, Hawaii.

## Abstract

**Introduction:**

Although glycemic control is known to reduce complications associated with diabetes, it is an elusive goal for many patients with diabetes. The objective of this study was to identify factors associated with sustained poor glycemic control, some glycemic variability, and wide glycemic variability among diabetes patients over 3 years.

**Methods:**

This retrospective study was conducted among 2,970 diabetes patients with poor glycemic control (hemoglobin A1c [HbA1c] >9%) who were enrolled in a health plan in Hawaii in 2006. We conducted multivariable logistic regressions to examine factors related to sustained poor control, some glycemic variability, and wide glycemic variability during the next 3 years. Independent variables evaluated as possible predictors were age, sex, type of insurance coverage, morbidity, diabetes duration, history of cardiovascular disease, and number of medications.

**Results:**

Longer duration of diabetes, being under age 35, and taking 15 or more medications were significantly associated with sustained poor glycemic control. Preferred provider organization and Medicare (vs health maintenance organization) enrollees and patients with high morbidity were less likely to have sustained poor glycemic control. Wide glycemic variability was significantly related to being younger than age 50, longer duration of diabetes, having coronary artery disease, and taking 5 to 9 medications per year.

**Conclusion:**

Results indicate that duration of diabetes, age, number of medications, morbidity, and type of insurance coverage are risk factors for sustained poor glycemic control. Patients with these characteristics may need additional therapies and targeted interventions to improve glycemic control. Patients younger than age 50 and those with a history of coronary heart disease should be warned of the health risks of wide glycemic variability.

## MEDSCAPE CME

Medscape, LLC is pleased to provide online continuing medical education (CME) for this journal article, allowing clinicians the opportunity to earn CME credit.

This activity has been planned and implemented in accordance with the Essential Areas and policies of the Accreditation Council for Continuing Medical Education through the joint sponsorship of Medscape, LLC and Preventing Chronic Disease. Medscape, LLC is accredited by the ACCME to provide continuing medical education for physicians. 

Medscape, LLC designates this Journal-based CME activity for a maximum of 1 **AMA PRA Category 1 Credit(s)™**. Physicians should claim only the credit commensurate with the extent of their participation in the activity.

All other clinicians completing this activity will be issued a certificate of participation. To participate in this journal CME activity: (1) review the learning objectives and author disclosures; (2) study the education content; (3) take the post-test with a 70% minimum passing score and complete the evaluation at www.medscape.org/journal/pcd (4) view/print certificate.

**Release date: September 26, 2012; Expiration date: September 26, 2013**

### Learning Objectives

Upon completion of this activity, participants will be able to:

- Analyze risk factors for poor glycemic control among patients with diabetes
- Evaluate risk factors for wide glycemic variability among patients with diabetes

**CME EDITOR**

Rosemarie Perrin, Editor; Camille Martin, Editor, *Preventing Chronic Disease*. Disclosure: Rosemarie Perrin and Camille Martin have disclosed no relevant financial relationships.

**CME AUTHOR**

Charles P. Vega, MD, Health Sciences Clinical Professor; Residency Director, Department of Family Medicine, University of California, Irvine. Disclosure: Charles P. Vega, MD, has disclosed no relevant financial relationships.

**AUTHORS AND CREDENTIALS**

Disclosures: Deborah Taira Juarez, ScD; Tetine Sentell, PhD; Sheri Tokumaru, PharmD, BCPS; Roy Goo, PharmD; James W. Davis, PhD; Marjorie M. Mau, MD, MS have disclosed no relevant financial relationships.

Affiliations: Deborah Taira Juarez, College of Pharmacy, University of Hawaii, Honolulu, Hawaii; Sheri Tokumaru, Roy Goo, University of Hawaii at Hilo, Hawaii; Tetine Sentell, James W. Davis, Marjorie M. Mau, John A. Burns, School of Medicine, Honolulu, Hawaii 

## Introduction

Risk of microvascular complications can be reduced by intensive glycemic control in patients with diabetes (1–5). The UK Prospective Diabetes Study (UKPDS), which followed participants for up to 10 years, found that intensive control (median hemoglobin A1c [HbA1c], 7.0%) reduced overall microvascular complication rates by 25% compared with conventional treatment. Possibly as a result of guidelines developed on the basis of this research, the percentage of diabetes patients with poor glycemic control (HbA1c >9%) decreased from 21.0% in 1999–2000 to 12.4% in 2003–2004 (6). Despite these encouraging trends, sustained glycemic control is an elusive goal for many patients with diabetes (1).

Wide glycemic variability may contribute to development of diabetic complications (7–9). One study found that diabetes patients who had episodes of both hypoglycemia and hyperglycemia were at greater risk of in-hospital mortality (10). Although evidence suggests a link between poor glycemic control and negative health outcomes among patients with diabetes (1–5), less is known about factors associated with achieving and sustaining glycemic control. Glycemic control is significantly associated with age, race/ethnicity, duration of diabetes, type and number of medications taken, obesity, psychological variables, and family support (11–22), although most studies examined cross-sectional associations. 

The objective of this longitudinal study was to identify characteristics associated with sustained poor glycemic control, some glycemic variability, and wide glycemic variability over 3 years among diabetes patients with initial poor glycemic control in 2006. 

## Methods

We conducted a retrospective analysis of administrative data from adult patients with diabetes who had poor glycemic control (HbA1c >9%) in 2006 and were enrolled in a large health plan in Hawaii. To be included in the study, patients needed to meet the following criteria: 1) be identified as having diabetes using algorithms employed by disease management programs; 2) be at least aged 18; 3) be enrolled with medical and drug coverage from 2006 through 2009; 4) have HbA1c measured at least once each year from 2006 through 2009; and 5) have at least 1 HbA1c measurement higher than 9% in 2006 (this level was chosen because the National Committee for Quality Assurance’s Healthcare Effectiveness Data and Information Set (HEDIS) uses a level of 9% to indicate poor glycemic control). Of the 4,667 patients with diabetes who had an HbA1c higher than 9% in 2006, 2,970 patients met the remaining criteria. Disease management algorithms were also used to identify patients with coronary artery disease and congestive heart failure. Diagnoses of diabetes, coronary artery disease, and congestive heart failure were confirmed whenever possible through contact of health plan enrollees and their physicians. A physician’s confirmation was required to exclude false positives.

During the baseline year (2006), we identified all patients with diabetes who had an HbA1c higher than 9%. For each subsequent year (2007–2009), we calculated mean HbA1c levels. We obtained patient information — age, sex, type of insurance coverage (health maintenance organization [HMO], preferred provider organization [PPO], Medicare cost contract), history of cardiovascular disease, number of medications, and morbidity level — from administrative data. History of coronary artery disease and congestive heart failure were modeled as dichotomous variables, with 1 indicating any history of either disease regardless of duration. Number of medications was divided into 4 categories (0–4, 5–9, 10–14, and ≥15). Patient morbidity level was determined by using *International Classification of Diseases, Ninth Revision, Clinical Modification* (*ICD-9-CM*) codes according to the Johns Hopkins Adjusted Clinical Groups methodology; levels of 4 or 5 on the 5-point scale were considered high morbidity (23). Duration of diabetes was calculated by subtracting the date of initial diabetes diagnosis in administrative claims, which was obtained from disease management files at the health plan, from the year of service (2006–2009). Duration was separated into 5 categories (1–3 y, 4–5 y, 6–7 y, 8–9 y, and ≥10 y).

For the 3-year period (2007–2009), we grouped patients into 4 categories: 1) *good control*, those who achieved and maintained a mean HbA1c of less than 7% for the 3 years (n = 166); 2) *poor control*, those who had a mean HbA1c higher than 9% for the 3 years (n = 2,034); 3) *wide glycemic variability*, those who had a reduction in annual mean HbA1c from higher than 9% to less than 7%, followed by an increase to higher than 9% during the 3 years (n = 76); and 4) *some glycemic variability*, those who did not meet criteria for any of the first 3 categories (ie, those who had some variability but did not meet our criteria of wide glycemic variability) (n = 694).

We used χ^2^ tests to assess differences in demographic variables (24). Separate multivariable logistic regression models were used to estimate the association between patient demographic variables and the likelihood of poor control, wide glycemic variability, and some glycemic variability. The comparison group was patients with good control (ie, patients initially in poor control who achieved and maintained good control from 2007 through 2009). Independent variables that were evaluated as possible predictors of glycemic control were age, sex, type of insurance coverage, morbidity level, diabetes duration, history of cardiovascular disease, and number of medications. Significance was set at α = .05. The University of Hawaii Committee on Human Studies approved this study as exempt. All analyses were conducted using Stata version 11.0 (StataCorp LP, College Station, Texas).

## Results

**Figure 1:**
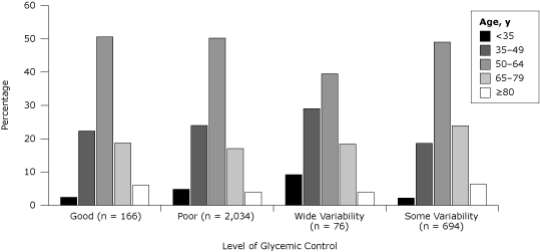
Glycemic control related to age in patients with diabetes, unadjusted, Hawaii, 2006–2009. Good control indicated by an HbA1c of less than 7% for 3 years, and poor control indicated by an HbA1c higher than 9% for 3 years. Wide glycemic variability refers to patients who had a reduction in annual mean HbA1c from higher than 9% to less than 7%, followed by an increase to higher than 9%. Some variability refers to patients who did not meet criteria for the other 3 categories. Glycemic control differed significantly by age (*P* < .001, Pearson χ^2^ tests)

The mean age of the study population was 57 years (standard deviation, 13 y) and 45.9% were female. Age, type of insurance coverage, history of coronary artery disease, diabetes duration, and number of medications differed significantly across groups defined by glycemic control (Table 1). Among patients with wide glycemic variability, 9.2% were under age 35, compared with 4.8% of patients with poor control and 2.4% of patients with good control (Figure 1). Of total patients, 21.9% with poor control were enrolled in the HMO, compared with 15.8% of patients with wide glycemic variability and 11.5% of patients with good control. The percentage of patients with a history of coronary artery disease ranged from 17.5% of patients with 3 years of good control to 29.1% of patients with some glycemic variability.

**Table 1:** Baseline Demographic Characteristics Associated With Glycemic Control^a^ for Adults with Diabetes (N = 2,970), Hawaii, 2006–2009

Characteristic	Good Control (n = 166), %	Poor Control (n = 2,034), %	Wide Glycemic Variability (n = 76), %	Some Glycemic Variability (n = 694), %	*P* Value^b^
**Female**	48.2	46.0	59.2	43.8	.07
**Type of insurance coverage**					
Health maintenance organization	11.5	21.9	15.8	20.3	<.001
Preferred provider organization	67.5	64.4	68.4	60.5	
Medicare cost contract	21.1	13.7	15.8	19.3	
**High morbidity^c^**	48.2	43.5	50.0	48.0	.12
**History of coronary artery disease**	17.5	25.0	27.6	29.1	.01
**History of congestive heart failure**	10.8	11.9	15.8	15.1	.11
**No. of medications^d^**					
≥15	13.3	24.6	26.3	23.0	<.001
10–14	16.3	21.8	7.9	22.2	
5–9	30.1	19.8	15.8	20.3	
0–4	40.4	33.9	50.0	34.3	

^a^ Good control indicates patients who achieved and maintained a mean HbA1c of less than 7% for 3 years (2007–2009) after the baseline year (2006); poor control indicates patients who had a mean HbA1c higher than 9% for 3 years after the baseline year; wide glycemic variability indicates patients who had a reduction in annual mean HbA1c from higher than 9% to less than 7%, followed by an increase to higher than 9% during the 3 years after the baseline year; and some glycemic variability indicates patients who did not meet criteria for any of the first 3 categories.

^b^ Pearson χ^2^ tests were used to determine whether there was a significant association between patient characteristics and glycemic control.

^c^ High morbidity was defined according to the Johns Hopkins Adjusted Clinical Groups methodology; levels of 4 or 5 on the 5-point scale were considered high morbidity (23).

^d^ Count of all medications taken by a patient during a given year.

**Table:** 

Age, y	Level of Glycemic Control				*P* Value
	Good (n = 166), %	Poor (n = 2,034), %	Wide Variability (n = 76), %	Some Variability (n = 694), %	
**<35**	2.4	4.8	9.2	2.2	<.001
**35–49**	22.3	24.0	29.0	18.6	
**50–64**	50.6	50.2	39.5	49.0	
**65–79**	18.7	17.0	18.4	23.9	
**≥80**	6.0	3.9	3.9	6.3	

**Figure 2:**
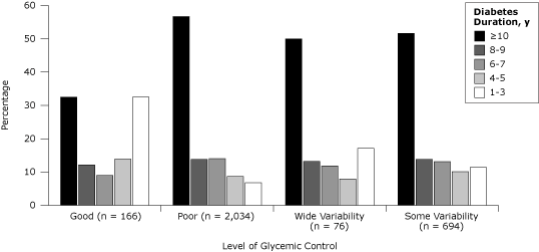
Glycemic control related to duration of diabetes in patients with diabetes, unadjusted, Hawaii, 2006–2009. Good control was indicated by an HbA1c of less than 7% for 3 years, and poor control was indicated by an HbA1c higher than 9% for 3 years. Wide glycemic variability refers to patients who had a reduction in annual mean HbA1c from higher than 9% to less than 7%, followed by an increase to higher than 9%. Some variability refers to patients who did not meet criteria for the other 3 categories. Glycemic control differed significantly by duration of diabetes (*P* < .001, Pearson χ^2^ tests).

Duration of time since the diagnosis of diabetes tended to be considerably lower for patients with good control with only 32.5% having had a diagnosis of diabetes for more than 10 years compared with over 50% in the sustained poor or variable groups (Figure 2). Similarly, the percentage of patients taking more than 15 medications was considerably lower for patients with good control at 13.3% compared with over 20% in the other groups. Sex, morbidity level, and history of heart disease did not differ significantly by level of glycemic control.

**Table:** 

Diabetes Duration, y	Level of Glycemic Control				*P* Value
	Good (n = 166), %	Poor (n = 2,034), %	Wide Variability (n = 76), %	Some Variability (n = 694), %	
**≥10**	32.5	56.7	50.0	51.6	<.001
**8–9**	12.1	13.8	13.2	13.8	
**6–7**	9.0	14.0	11.8	13.11	
**4–5**	13.9	8.7	7.9	10.1	
**1–3**	32.5	6.8	17.1	11.4	

### Factors associated with poor control

A total of 68.5% (n = 2,034) of patients had poor glycemic control for all 3 years following the baseline year. Compared with patients aged 50 to 64, patients aged less than 35 years were significantly more likely to have poor control (Table 2). Compared with HMO enrollees, patients enrolled in the PPO or the Medicare cost contract were significantly less likely to have poor control. Similarly, patients with high morbidity were significantly less likely to have poor control after adjustment for other factors.

**Table 2:** Characteristics Associated with Variable Glycemic Control in Adult Patients with Diabetes, Relative to Patients With Good Control,^a^ Hawaii, 2006–2009

Characteristic	Poor Control (n = 2,034), OR (95% CI)	Wide Glycemic Variability (n = 76), OR (95% CI)	Some Variability (n = 694), OR (95% CI)
**Age**, **y**			
<35	2.90 (1.02–8.22)	7.67 (1.71–34.52)	1.72 (0.46–6.38)
35–49	1.37 (0.89–2.10)	2.55 (1.15–5.68)	1.05 (0.64–1.71)
50–64	1 [Reference]	1 [Reference]	1 [Reference]
65–79	1.33 (0.69–2.56)	1.40 (0.52–3.79)	1.68 (0.83–3.42)
≥80	1.03 (0.40–2.68)	1.21 (0.24–6.17)	1.49 (0.54–4.10)
**Sex**			
Female	0.97 (0.69–1.37)	1.77 (0.95–3.30)	0.81 (0.56–1.18)
Male	1 [Reference]	1 [Reference]	1 [Reference]
**Type of insurance coverage**			
Health maintenance organization	1 [Reference]	1 [Reference]	1 [Reference]
Preferred provider organization	0.44 (0.26–0.75)	0.58 (0.22–1.48)	0.44 (0.25–0.78)
Medicare cost contract	0.28 (0.12–0.62)	0.30 (0.08–1.19)	0.23 (0.10–0.56)
**Morbidity level**			
Low	1 [Reference]	1 [Reference]	1 [Reference]
High^b^	0.62 (0.44–0.87)	0.80 (0.40–1.57)	0.69 (0.47–1.01)
**History of coronary artery disease**			
Yes	1.50 (0.89–2.53)	2.42 (1.01–5.82)	1.61 (0.97–2.69)
No	1 [Reference]	1 [Reference]	1 [Reference]
**History of congestive heart failure**			
Yes	0.73 (0.39–1.34)	1.06 (0.34–3.26)	0.90 (0.48–1.71)
No	1 [Reference]	1 [Reference]	1 [Reference]
**Diabetes duration, y**			
≥10	9.20 (5.82–14.54)	3.48 (1.46–8.32)	4.76 (2.90–7.81)
8–9	5.21 (2.94–9.24)	1.79 (0.59–5.43)	3.17 (1.69–5.95)
6–7	7.20 (3.84–13.51)	3.00 (1.02–8.82)	4.08 (2.09–7.98)
4–5	2.91 (1.66–5.12)	1.14 (0.38–3.46)	2.18 (1.18–4.02)
1–3	1 [Reference]	1 [Reference]	1 [Reference]
**No of medications^c^**			
≥15	2.08 (1.23–3.54)	1.54 (0.65–3.69)	2.10 (1.16–3.81)
10–14	1.58 (0.95–2.63)	0.42 (0.15–1.18)	0.84 (0.52–1.34)
5–9	0.75 (0.49–1.15)	0.37 (0.15–0.90)	0.84 (0.53–1.34)
0–4	1 [Reference]	1 [Reference]	1 [Reference]

Abbreviations: OR, odds ratio; CI, confidence interval.

^a ^Poor control indicates patients who had a mean HbA1c higher than 9% for 3 years (2007–2009) after the baseline year (2006); wide glycemic variability indicates patients who had a reduction in annual mean HbA1c from higher than 9% to less than 7%, followed by an increase to higher than 9% for 3 years after the baseline year; some glycemic variability indicates patients who did not meet criteria for any of the first 3 categories. Patients with good control achieved and maintained a mean HbA1c of less than 7% for 3 years after the baseline year.

^b^ High morbidity was defined according to the Johns Hopkins Adjusted Clinical Groups methodology; levels of 4 or 5 on the 5-point scale were considered high morbidity (23).

^c^ Count of all medications taken by a patient during a given year.

The likelihood of poor control increased with diabetes duration (Table 2). Compared with patients who had diabetes for 3 years or less, patients with diabetes for 10 or more years were more than 9 times as likely to have poor control. Patients taking 15 or more medications were more likely to have poor control compared with patients taking fewer than 5 medications, although the likelihood of poor control for patients taking 5 to 14 medications did not significantly differ. In the adjusted model, sex and history of coronary artery disease and congestive heart failure were not significantly associated with poor control.

### Factors associated with glycemic variability

During the 3 follow-up years, 2.6% (n = 76) of the study population had wide glycemic variability, which was significantly associated with age, history of coronary artery disease, duration of diabetes, and number of medications (Table 2). Compared with patients aged 50 to 64, patients aged less than 35 and aged 35 to 49 were significantly more likely to have wide glycemic variability. Longer duration of diabetes was associated with wide glycemic variability (Table 2); patients with diabetes for 6 to 7 years or for 10 years or more were more likely to have wide glycemic variability than patients with diabetes for 3 years or less. Patients taking 5 to 9 medications (vs <5 medications) were significantly less likely to have wide glycemic variability (Table 2). Sex, type of insurance coverage, morbidity level, and history of congestive heart failure, were not significantly related to wide glycemic variability. 

Patients with PPO or Medicare coverage were significantly less likely to have some, but not wide, glycemic variability than patients enrolled in the HMO (Table 2). In contrast, longer duration of diabetes was associated with having some variability in glycemic control, as was use of 15 or more medications.

## Discussion

Knowledge of factors influencing glycemic control can be used by health professionals to provide targeted interventions to patients at greatest risk of diabetic complications. We studied almost 3,000 diabetes patients enrolled in a large health plan in Hawaii who had an initial HbA1c higher than 9% in 2006 and examined factors associated with sustained poor glycemic control, some glycemic variability, and wide glycemic variability over the subsequent 3 years. Longer duration of diabetes, taking 15 or more medications per year, being enrolled in the HMO, and being under age 35 were significantly associated with sustained poor glycemic control, and having a high morbidity level was negatively associated with sustained poor control.

Our findings are consistent with those of previous studies in that older patients tended to achieve better control (11–14) and that the duration of diabetes (13–15) and number of medications (12,17) were associated with poor control. Therefore, factors previously associated with glycemic control in cross-sectional studies are similar to those related to sustained control over 3 years for patients who initially had poor control. It is not surprising that poor glycemic control is significantly associated with longer duration of diabetes and larger number of medications, given that diabetes is a progressive disease and that, as glucose levels rise, more drugs are required to try to achieve better control.

To our knowledge, our study is the first to examine factors predicting wide glycemic variability over a 3-year period. Patients who are capable of achieving good HbA1c levels, only to revert back to poor glycemic control, may be a subgroup that could benefit from a targeted intervention to maintain the good control once achieved. Prior research has shown that good glycemic control at 7% or less for at least 3 years’ duration may have long-term benefits for approximately 8 years, during which microvascular benefits of glycemic control have extended benefits (“metabolic memory”) (25). This group with good glycemic control for a short period is incurring all the additional cost of medications and physician visits but could potentially realize a greater benefit if they were able to maintain good glycemic control for a longer period.

Few published intervention studies have focused on addressing challenges faced by younger adult patients with diabetes, and those that have often have not produced significant results (26,27). One approach that has shown some success among younger adults has been professionally led support groups to increase self-motivation and facilitate peer-to-peer interactions (28). Moreover, as evidence suggests that high consumption of fat and sugar is closely associated with elevations in HbA1c levels among those aged less than 55 years (29), young adults may benefit from interventions focused on diet.

Patients with HMO insurance were more likely to sustain poor glycemic control and to have variable control compared with PPO- or Medicare-insured patients. The cause of this disparity is unclear, because the health plan’s disease management program does not differ by type of insurance coverage, and drug coverage for PPO and HMO members is similar. It may be a selection issue, because the HMO tends to require lower out-of-pocket payments for inpatient services but higher copayments for physician office visits, or a difference in socioeconomic characteristics related to type of insurance coverage that was not captured in our data set. Further research is needed to better understand the effect of type of insurance coverage on reaching good glycemic control and maintaining it.

There are several limitations to this study. First, patients were enrolled in a large health plan in Hawaii, so results may not be generalizable to other geographic areas or to uninsured patients. Second, to be included in the study, patients had to have 4 years of both medical and drug coverage as well as HbA1c screening, which may have resulted in a select group of patients who may not be representative of all people with diabetes who are in poor control in a given year. Third, our study did not include information on other variables, including obesity, psychological characteristics, race/ethnicity, smoking, and physician specialty, that are known to be associated with glycemic control (18–22). Fourth, we did not examine processes of care related to glycemic control, such as medication regimens that may affect diabetes medication adherence. Although we knew the number of medications, we did not have detailed data on patients’ medication therapy. Further research is needed to determine the extent to which poor glycemic control is associated with deviations from evidence-based guidelines for the achievement of glycemic control (30). For instance, insulin may have been underused in this study population.

Despite these limitations, our study offers new insight into subgroups of patients with sustained poor glycemic control and wide glycemic variability who may benefit from targeted interventions. In particular, younger adult patients (eg, less than age 35, aged 35–49) may be a good target population because of their high odds of poor control and the fact that they will have more years to live with diabetes. Further research is needed to determine what interventions work best for these younger patients, although existing evidence suggests that professionally led peer-to-peer interactions and interventions that target diet may be appropriate and effective. Wide glycemic variability among patients under age 50, particularly among those under age 35, and with a history of coronary artery disease indicates these patients may need further education or programs to reduce this variability. This subset of patients may require a modified approach to good glycemic maintenance strategies that are distinct from patients who were not able to achieve good glycemic control throughout the 3-year period. We hope that findings from this study will assist clinicians, health plans, disease management vendors, and others in developing targeted interventions for subgroups of patients with poorly controlled diabetes.

## Figures and Tables

**Table:** 

**1. The activity supported the learning objectives.**				
**Strongly Disagree**				**Strongly Agree**
1	2	3	4	5
**2. The material was organized clearly for learning to occur.**				
**Strongly Disagree**				**Strongly Agree**
1	2	3	4	5
**3. The content learned from this activity will impact my practice.**				
**Strongly Disagree**				**Strongly Agree**
1	2	3	4	5
**4. The activity was presented objectively and free of commercial bias.**				
**Strongly Disagree**				**Strongly Agree**
1	2	3	4	5

## Post-Test Information

To obtain credit, you should first read the journal article. After reading the article, you should be able to answer the following, related, multiple-choice questions. To complete the questions (with a minimum 70% passing score) and earn continuing medical education (CME) credit, please go to http://www.medscape.org/journal/pcd. Credit cannot be obtained for tests completed on paper, although you may use the worksheet below to keep a record of your answers. You must be a registered user on Medscape.org. If you are not registered on Medscape.org, please click on the "Register" link on the right hand side of the website to register. Only one answer is correct for each question. Once you successfully answer all post-test questions you will be able to view and/or print your certificate. For questions regarding the content of this activity, contact the accredited provider, CME@medscape.net. For technical assistance, contact CME@webmd.net. American Medical Association's Physician's Recognition Award (AMA PRA) credits are accepted in the US as evidence of participation in CME activities. For further information on this award, please refer to http://www.ama-assn.org/ama/pub/category/2922.html. The AMA has determined that physicians not licensed in the US who participate in this CME activity are eligible for **AMA PRA Category 1 Credits™**. Through agreements that the AMA has made with agencies in some countries, AMA PRA credit may be acceptable as evidence of participation in CME activities. If you are not licensed in the US, please complete the questions online, print the AMA PRA CME credit certificate and present it to your national medical association for review.

## Post-Test Questions

### Article Title: RF for Poor Glycemic Control

**CME Questions**

1. You are seeing a 57-year-old woman new to your practice. She has a history of type 2 diabetes mellitus with highly irregular follow-up with her previous physician. The patient is afraid that her diabetes is not well controlled and has several questions about risk factors for poor glycemic control. Which of the following was a risk factor for poor glycemic control in the current study?
  - Female sex
  - Age over 50 years
  - A history of congestive heart failure
  - The presence of multiple comorbid conditions
2. As you talk with this patient, what was the type of insurance was associated with the highest risk of poor glycemic control in the current study?
  - No insurance/cash pay
  - HMO
  - PPO
  - Medicare
3. What was the relationship between the duration of diabetes and the risk of poor glycemic control in the current study?
  - The risk of poor glycemic control was inversely related to the duration of diabetes
  - The risk of poor glycemic control was particularly elevated among individuals with diabetes for 5 years or less
  - The risk of poor glycemic control was particularly elevated among individuals with diabetes for 10 years or more
  - There was no significant interaction between the duration of diabetes and the risk of poor glycemic control
4. The patient returns to your clinic one month later with widely variable home glucose readings. Which of the following variables was a risk factor for wide glycemic variability in the current study?
  - Taking fewer than 5 medications regularly
  - Age over 50 years
  - HMO insurance
  - Higher number of comorbid diseases

**Evaluation**
